# Supplementary material for: Basic reproduction number varies markedly between closely related pandemic Escherichia coli clones
Source: Nat Commun. 2025 Nov 4;16:9490. doi: 10.1038/s41467-025-65301-1 (PMC12586494; doi:10.1038/s41467-025-65301-1)
Supplement: Supplementary file 1 — Supplementary Information [file 41467_2025_65301_MOESM1_ESM.pdf]

Supplementary Information for Basic reproduction number varies markedly between closely related pandemic *Escherichia coli* clones

Fanni Ojala, Henri Pesonen, Rebecca A. Gladstone,
Tommi Mäklin, Gerry Tonkin-Hill, Pekka Marttinen,
Jukka Corander

*Corresponding author(s). E-mail(s): jukka.corander@medisin.uio.no;

1 Sensitivity analysis

As sensitivity analysis, we examined the impact of two parameters, relative invasiveness ρ_c and the delay parameter Δ_t on the posterior distribution of R_0 and the fit of the model to data. Since the relative invasiveness (odds ratio) ρ_c was an estimate itself, we chose to use the 95% confidence intervals for ρ_c as sensitivity analysis: the model was fit for the lower 95% CI, mean value and upper 95% CI estimate. The mean value of ρ_c was used for the main results.

In addition, we tested the impact of different priors for the delay parameter Δ_t, which defines the delay between onset of colonization and infection. The prior is uniform in all cases, but three different maximum values for Δ_t were tested: 0 years (no delay), 0.5 years and 5 years. A maximum delay of 0.5 years was used in the main results.

In addition to the posterior distribution of R_0, we assess the sensitivity of our model to ρ_c and Δ_t in terms of model fit. Supplementary Figures 2 and 3 show a visual comparison between different choices of ρ_c and Δ_t.

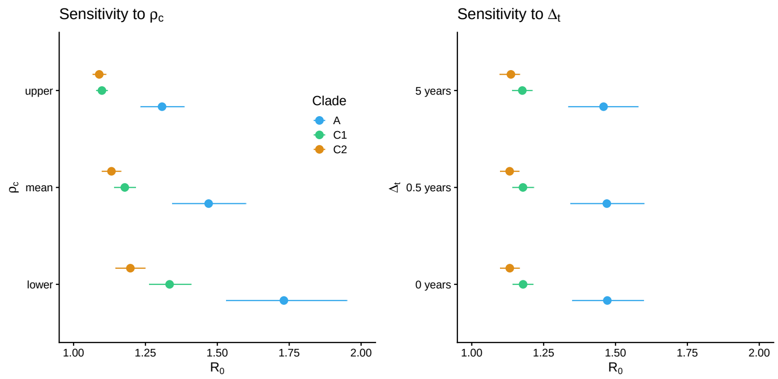

Fig. 1 Impact of ρ_c and Δ_t on the R_0 posterior distribution as a forest plot with 95% credible intervals as a horizontal line and the mean estimate as a point. On the left hand side, we display the effect of different ρ_c estimates on the posterior distribution of R_0, colored by clade. On the right hand side, we display the 95% credible interval for R_0 for three different maximum delays: 0 years, 0.5 years and 5 years. Results for all clades seem identical regardless of delay value.

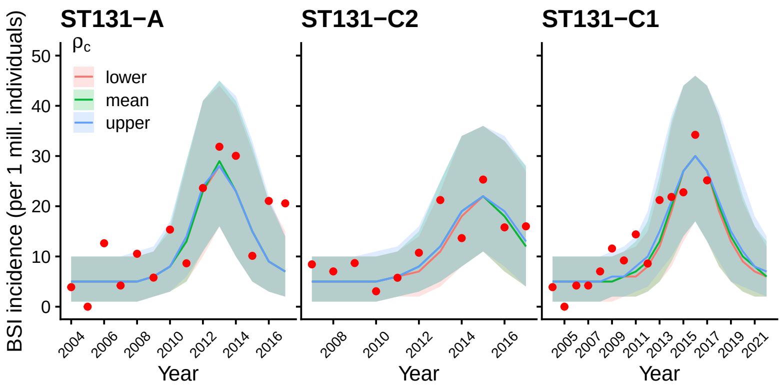

Fig. 2 Effect of different ρ_c estimates on the model fit to data. The different colors correspond to data fits using different ρ_c estimates: lower and upper 95% CI and the mean ρ_c estimate, which was used for the main results. The red dots correspond to observed data.

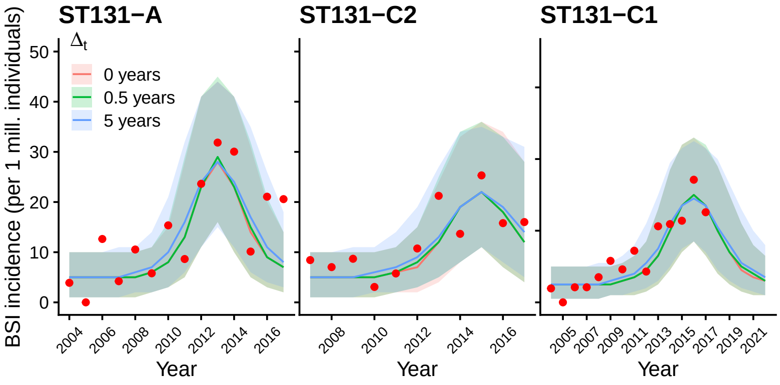

Fig. 3 Effect of different maximum Δ_t values on the posterior predictive model fit to data. The red dots correspond to observed data. We can observe a slight increase in uncertainty as the maximum of the delay parameter is increased to 5 years.

2 Model comparison

We compare the chosen colonization model (SIR-based compartmental model), with an alternative SIS-based compartmental model. The SIS-based model does not assume a recovered/removed compartment and thus allows for re-colonization. For model comparison, we used ABC-SMC thresholds of 2.0, 1.5, 1.0 and 0.5 and locally optimal proposal distributions to allow for faster inference, since the SIS model was considerably more challenging to fit to the data. For C2 clade the simulation process was extended to 14 years due to difficulties of generating sufficiently many accepted particles from the posterior under the SIS model due to its poorer fit to data. For all clades, the acceptance rate of the SIS-based model is considerably lower for the smallest threshold of 0.5 than the acceptance rate for the SIR-based model. For ST131-C2, the decrease in acceptance rate starts is most notable, from threshold value of 1.0 to 0.5, as seen in Figure 5. From Figure 4 it can be seen that the fit to data is quite poor, especially for ST131-C2 and ST131-C1.

Table 1 Acceptance rate (number of samples divided by number of simulations), number of samples, number of simulations and the threshold value for SIR and SIS models. Models were run for clades ST131-A, ST131-C2 and ST131-C1. Note that for ST131-A and ST131-C2 a locally optimal ABC-SMC algorithm was used.

ST131	SIR Acceptance rate	SIS Acceptance rate	Threshold
A	0.1236	0.1643	2.0
	0.1360	0.0961	1.5
	0.0414	0.0444	1.0
	0.1204	0.0282	0.5
C2	0.0943	0.0610	2.0
	0.0806	0.0506	1.5
	0.0146	0.0080	1.0
	0.0027	0.0001	0.5
C1	0.0498	0.0358	2.0
	0.0350	0.0137	1.5
	0.0086	0.0018	1.0
	0.0071	0.0007	0.5

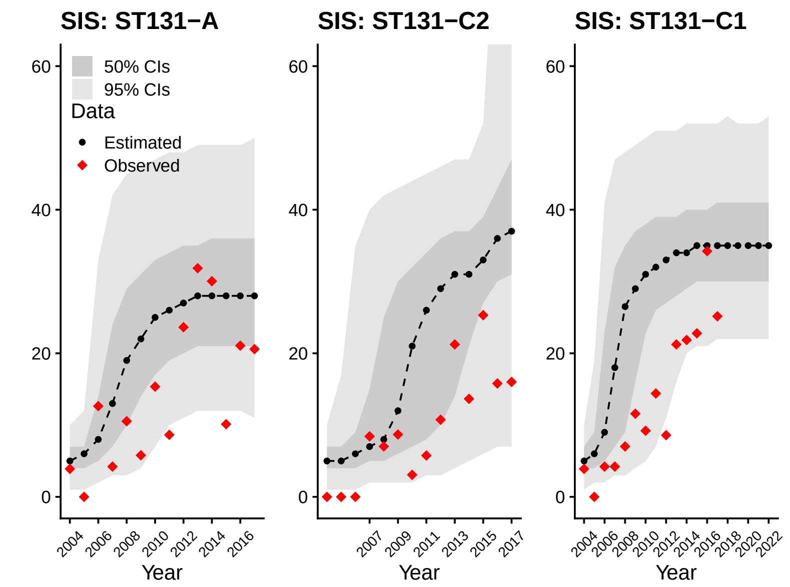

Fig. 4 Fit of the SIS model to data. Red diamonds depict the observed data and black dots represent the mean of the estimated data. Grey and dark grey area are the 95 and 50 % credible intervals.

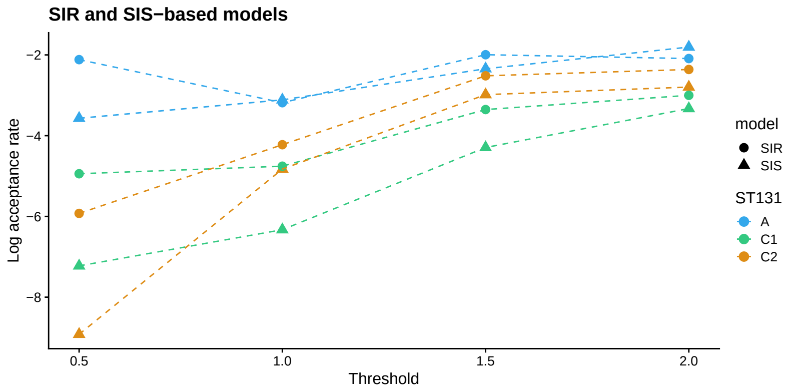

Fig. 5 Logarithmic (natural logarithm) acceptance rate as a function of threshold for clades ST131-A, ST131-C2 and ST131-C1. Triangle-shaped points correspond to the SIS-based model and circular points to the SIR-based model.

3 Other Supplementary Items

3.1 Identifiability of R_0, τ and Δ_t

Plots of overlaid prior and posterior distributions of model parameters for clades ST131-A, ST131-C2 and ST131-C1 show that R_0 and τ are well identifiable, while the delay parameter Δ_t is not.

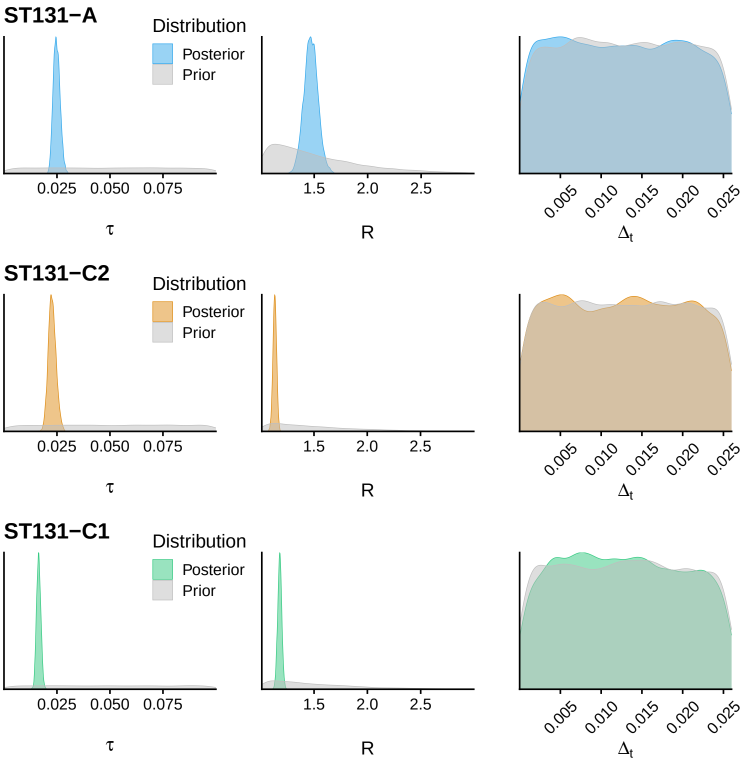

Fig. 6 Overlay of the prior and posterior distributions for the estimated parameters τ, R_0 and Δ_t for the clades of interest.

3.2 Duration of colonization

In Figure 7, we show the posterior distribution of the duration of colonization for ST131-A, ST131-C2 and ST131-C1 in weeks. The duration of colonization is defined as $1/\gamma$, where γ is the recovery rate of an SIR model. Refer to the main text for details on the model parametrization.

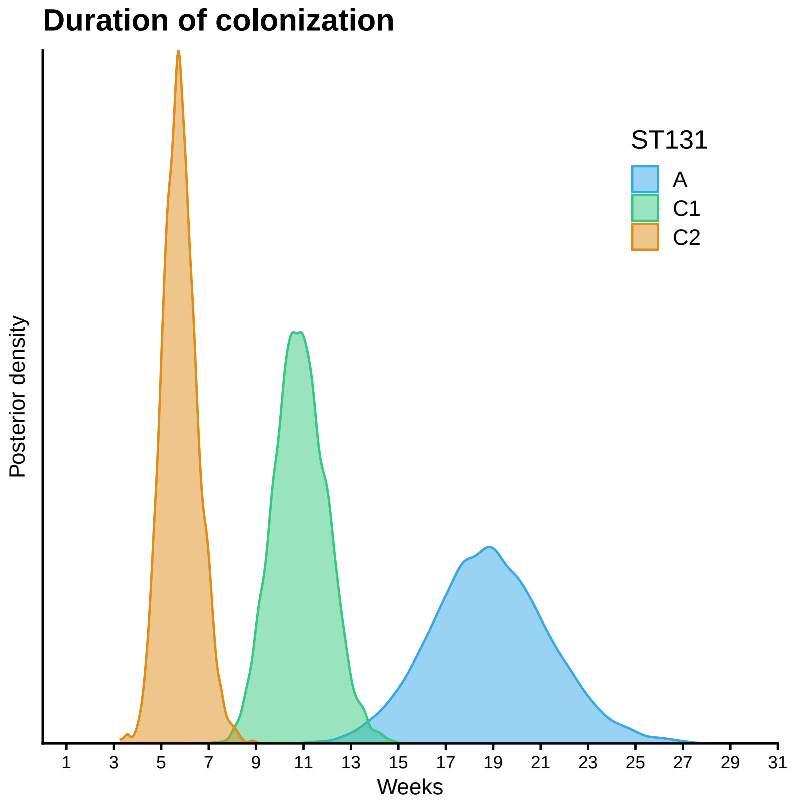

Fig. 7 Posterior distribution of the duration of colonization in weeks, defined as $1/\gamma$, where γ is the recovery rate of an SIR model.
